# Berberine influences multiple diseases by modifying gut microbiota

**DOI:** 10.3389/fnut.2023.1187718

**Published:** 2023-08-03

**Authors:** Fujie Yang, Rongmao Gao, Xiaoxiu Luo, Rongan Liu, Daqian Xiong

**Affiliations:** ^1^Department of Laboratory Medicine, Hospital of Chengdu University of Traditional Chinese Medicine, Chengdu, China; ^2^College of Medical Technology, Chengdu University of Traditional Chinese Medicine, Chengdu, China; ^3^Department of ICU, Sichuan Provincial People’s Hospital, University of Electronic Science and Technology of China, Chengdu, China

**Keywords:** berberine, gut microbiota, metabolic diseases, liver disease, intestinal diseases, autoimmune diseases

## Abstract

Berberine (BBR) is an isoquinoline alkaloid that is widely distributed in the plant kingdom and is commonly found in *Coptis chinensis* Franch. It has low bioavailability, but it can interact with gut microbiota and affect a variety of diseases. The effects of BBR in diabetes, hyperlipidemia, atherosclerosis, liver diseases, intestinal diseases, mental disorders, autoimmune diseases, and other diseases are all thought to be related to gut microbiota. This review systematically and comprehensively summarize these interactions and their effects, and describes the changes of gut microbiota after the intervention of different doses of berberine and its potential clinical consequences, in order to provide a basis for the rational application of BBR in the future clinical treatment.

## 1. Background

Berberine (BBR) is able to be extracted from the roots and rhizomes of a variety of medicinal plants, such as *Berberis kansuensis* C.K. Schneid. (Berberidaceae), *Coptis chinensis* Franch. (Ranunculaceae), *Coscinium fenestratum* (Goetgh.) Colebr. (Menispermaceae), *Argemone mexicana* L. (Papaveraceae), and *Phellodendron amurense* Rupr. (Rutaceae),whose chemical composition is an isoquinoline alkaloid (1). It is used to treat a wide range of diseases, including tumor, endocrine diseases, cardiovascular diseases, neurological diseases, and digestive diseases (2). Animal and clinical studies have demonstrated that BBR promotes insulin secretion, increases insulin sensitivity, inhibits gluconeogenesis, reduces lipid accumulation, inhibits steatosis and fibrosis, has properties that reduce inflammatory responses and oxidative stress, and modulates the immune system [(1, 3, 4)]. Recent advancements in microbial sequencing technology, metabolomics technology, and sterile animal models have focused attention on the study of intestinal microecology (5). The gut microbiota plays a crucial role in the digestion and absorption of nutrients, metabolism, immune function, and disease development in the host. Maintaining the stability of the intestinal microecological environment is essential for regulating the health of the host. So it is important to keep the intestinal micro-ecological environment stable for controlling the health of the host. In recent years, there is growing evidence that BBR can reverse the composition and value of gut microbiota in non-healthy state (Tables 1, 2). Hyperlipidemia, diabetes, cancer, and inflammatory diseases suggest an key correlation between gut microbiota and BBR (6). But the target of BBR needs to be further investigated. With low oral bioavailability, it may influence gut microbiota. It is not thoroughly understood what the results of BBR for gut microbiota is and how altered flora relate to the metabolic benefits of BBR. This paper aims to review the role of gut microbiota under pathological conditions after BBR treatment in the background of fundamental studies and application of BBR in clinical, described the modulatory effects of BBR on the composition of the gut microbiota and its metabolites, and discussed the interaction between gut microbiota and BBR to provide more support for basic research and clinical trials.

**TABLE 1:** Berberine regulates gut microbiota in animal models.

Bioassay model	Dosage	Key finding	References
diabetic db/db mice	136.5 mg/kg, i.g. for 11 weeks	↑SCFAs-producing bacteria	(11)
diabetic rats	200 mg/kg, i.g. 22 for weeks	↑Butyrate-producing bacteria	(13)
diabetic rats	200 mg/kg, i.g. for 6 weeks	↑*Bacteroides*,*Lactobacillaceae*;↓ Proteobacteria, *Verrucomicrobia*	(14)
diabetic db/db mice	BBR (210 mg/kg), oryzanol (33.6 mg/kg),vitamin B6 (7 mg/kg), for 4 weeks	↑BSH-producing bacteria	(17)
diabetic rats	100 mg/kg, p.o. for 30 days + 150 mg/kg, p.o.for 18 days	↑*Akkermansiaceae*, *Erysipelotrichaceae*, *Desulfovibrionaceae*; ↓ *Enterobacteriaceae*, *Christensenellaceae*, *Bifidobacteriace*	(18)
Hyperlipidemic rats	150 mg/kg,p.o. for 16 weeks	↓*Firmicutes*	(22)
NAFLD rats	200 mg/kg. i.g. for 8 weeks	↑*Bifidobacteria*	(25)
NAFLD rats	150 mg/kg. i.p. for 4 weeks	↑*Bacteroides;*↓*Faecalibacterium prausnitzii*	(26)
NASH mice	100 mg/kg. i.g. for 4 weeks	↑*Clostridiales*, *Lactobacillaceae, Bacteroidale*	(27)
ALD mice	10, 50, 100 mg/kg. i.g. for 33 days	↑*Akkermansia muciniphila*↓*Pseudoflavonifractor, Mucisirillum, Alistipes, Ruminiclostridium, Lachnoclostridium*	(28)
AS mice	100, 200 mg/kg, p.o. for 4 months	↑*Lachnospiraceae NK4A136group, Bacteroidales S24-7 group (unclassified), Eubacterium*	(33)
AS mice	50 mg/kg, i.g. for 12 weeks	↑*Verrucomicrobia;*↓*Firmicutes*	(35)
AS mice	0.5 g/L, p.o. for 14 weeks	↑*Akkermansia*	(36)
Colitis rats	40 mg/kg, i.g. for 7 days	↑*Bacteroides*,*Akkermansia*	(41)
Colitis mice	40 mg/kg, i.g. for 7 days	↑*Lactococcus*;↓*Mouse intestinal Bacteroides*, *Segmented filamentous bacteria*,*Enterobacteriaceae*	(42)
UC mice	40 mg/kg, i.g. for 7 days	↑Lactic acid-producing bacteria, carbohydrate hydrolysis bacteria;↓conditional pathogenic bacteria	(43)
UC mice	100 mg/kg, p.o. for 8 days	↑*Bacteroides fragilis*	(44)
IBS rats	200 mg/kg, p.o. for 14 days	↑SCFAs-producing bacteria;	(47)
IBS rats	BA-BBR 1.715 mg, i.g. for 10 days	↓ *Bacteroidia, Deferribacteres, Verrucomicrobia, Candidatus_Saccharibacteria, Cyanobacteria*	(48)
CRC mice	100 mg/kg, p.o. for 10 weeks	↑SCFAs-producing bacteria;↓*f_Erysipelotrichaceae, Alistipes*	(52)
CRC Apc^min/+^ mice	500 ppm, p.o. for 12 weeks	↑*Lachnospiraceae;*↓*Akkermansia*	(53)
CRC mice	7.5, 15 mg/kg, i.g. for 4 weeks	↑*Lactobacillus,Dubosiella;*↓*Bacteroides,* *Escherichia-Shigella,Akkermansia*	(54)
Anxiety rats	100 mg/kg, i.g. for 4 weeks	↑*Bacteroides, Bifidobacterium, Lactobacillus, Akkermansia*	(62)
EAU mice	100 mg/kg, i.g. for 2 weeks	↑*Lactobacillus,Akkermansia,Oscillibacter,* *Ruminocococaceae*	(67)
GVHD mice	50 mg/kg, i.g. for 25 days	↑Actinobacteria, Bacteroidetes, *Adlercreutzia*, *Lactobacillus*, *Dorea*, *Sutterella*,*Plesiomonas*	(68)
Allograft mice	200 mg/kg, i.g. for 3 weeks	↓*Bacillus cereus*	(69)
CIA mice	200 mg/kg, i.g. for 2 weeks	↑SCFAs-producing bacteria	(70)
PD mice	100, 200 mg/kg, i.g. for 24 h	*Enterococcus* might be an interesting genus for dopa/dopamine biosynthesis in the intestine, and BBR might promote the body dopa/dopamine levels through the bacteria in intestine.	(71)
Periodontitis rats	120 mg/kg, i.g. for 7 weeks	↑Butyrate-producing bacteria	(72)

**TABLE 2:** Changes of gut microbiota in clinical subjects regulated by berberine.

Bioassay model	Dosage	Key finding	References
Hyperglycemia patients	0.5 g, bid, p.o. for 16 weeks	↑*Blautia;*↓*Roseburia*	(15)
T2DM patients	3.6 g, bid, p.o. for 12 weeks	BBR is mediated by the inhibition of DCA biotransformation by *Ruminococcus bromii*	(16)
Hyperlipidemic patients	0.5 g, bid, p.o. for 12 weeks	the baseline abundance of *Alistipes* and *Blautia* could effectively predict the cholesterol-decreasing efficacy of BBR	(21)
postprandial lipidemia patients	BBR (0.6 g per 6 pills, bid) + probiotics (4 g per 2 strips of powder, qd), p.o. for 12 weeks	*Bifidobacterium breve* and BBR could exert a synergistic hypolipidemic effect on postprandial lipidemia	(24)
Schizophrenia or bipolar disorder patients	100–300 mg/tid, p.o. for 12 weeks	↑*Bacteroides*;↓*Firmicutes*	(63)
Graves patients	300 mg/tid, p.o. for 24 weeks	↑*Lactococcus lactis;*↓*Enterobacter hormaechei, Chryseobacterium indologenes*	(66)

## 2. Methods

Two authors searched the literature published in 2022 06 through MEDLINE (PubMed), EMBASE. Using “berberine” and “gut microbiota” as keywords, they included the literature meeting the following criteria: patients and animal models of gut microbiota were studied; studies were clinical trials or animal experiments of berberine intervention; and the primary endpoints were changes in organ function, metabolic status, and inflammatory response. Authors conducted this study in three stages: analyzing the title followed by the abstract and, finally, reading the full text in detail. They were able to retrieve 170 articles from PubMed and 286 (with 3 duplicates) from EMBASE, for a total of 124 duplicates in both databases 260 irrelevant articles were excluded by title, and 3 systematic reviews were excluded after reading the abstracts. 16 non-disease studies, and 15 low-quality literature pieces were also excluded. Finally, 35 studies were included after reading the full text. The literature had to meet the following criteria: all clinical and basic studies on diseases connected with intervention of gut microbiota through the BBR active ingredient pathway, and the language of the literature was limited to English. There were no BBR-related compounding agents involved in the study.

## 3. Effect of BBR on gut microbiota in different diseases

### 3.1. Diseases related to glycolipid metabolism

Glycolipid metabolic diseases are common chronic diseases in the clinic that have been attracting increasing attention. Approximately 1.5 billion people worldwide have metabolism-related diseases, making it a global public health issue (7).

It is important for BBR in treating these diseases by affecting the gut microbiota according to the latest research. This promotes insulin secretion, improves insulin resistance, and inhibits lipogenesis which is associated with changes in the composition of the gut microbiota and its metabolites [(1, 8, 9)].

#### 3.1.1. Diabetes mellitus

**FIGURE 1:**
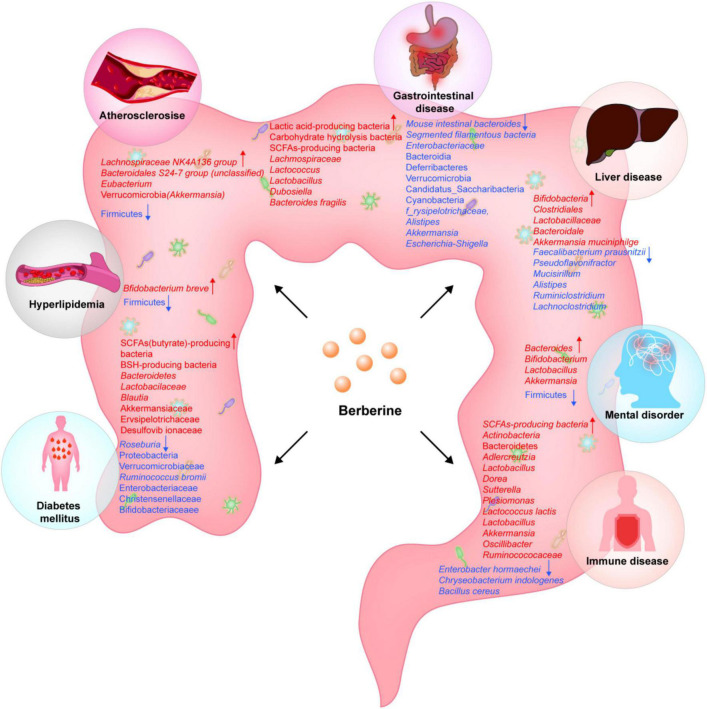
Berberine affects the changes of gut flora in different diseases.

Modern pharmacological studies show the importance of gut microbiota in the developmental phases of type 2 diabetes mellitus (T2DM) similar to that of genetic-, environmental-, and dietary factors (9, 10). Berberine is intragastric in db/db mice with the dosage of 136.5 mg/kg, and the proportion of *Butyricimonas*, *Coprococcus*, and *Ruminococcus* bacteria producing short-chain fatty acids (SCFAs) increases (11) (Figure 1). Short chain fatty acids cause an increase in glucagon-like peptide-1 (GLP-1) secretion, enhance insulin secretion and suppress glucagon secretion to improve blood glucose levels (12). Gegen Qinlian decoction (containing BBR as the key component) and BBR (200 mg/kg, 22 weeks) alone enrich butyrate-producing bacteria, such as *Faecalibacterium* and *Roseburia*, and increase the level of SCFAs in the feces (13).

In a rat model of diabetes, intragastric administration of berberine at a dosage of 200 mg/kg suppressed blood glucose levels, improved glucose tolerance, and serum lipid parameters after 6 weeks. The relative abundance of Bacteroides increases in the BBR group, while the relative abundance of Proteobacteria and Verrucomicrobia phyla decreases. Probiotic *Lactobacillaceae* are significantly up-regulated in the BBR group and have a negative correlation with the risk of T2DM (14).

The combined application of BBR and probiotics shows that BBR regulates the structure and action of gut microbiota, and *Bifidobacterium* potentially enhances the hypoglycemic effect of BBR (15) (There is a significant reduction in blood glucose in the group of BBR and the group of BBR- *Bifidobacterium* combination after 16 weeks of treatment with oral BBR (0.5 g twice daily) at 2 h postprandial compared with that in the control. Both groups show reduced abundance of intestinal bacteria *Roseburia*, including *Ruminococcus gnavus* and *Ruminococcus*, while the abundance of *Blautia* increases. In addition, the abundance of Proteobacteria dramatically increases in the BBR group, but not in the combination group, which may be related to the use of *Bifidobacterium* (15). A randomized double-blind controlled trial enrolling 409 patients with T2DM receiving probiotics, BBR (0.6 g/6 capsules twice daily), probiotics + BBR, or placebo for 12 weeks after 1 week of gentamicin pretreatment shows that glycated hemoglobin is more significantly altered in the probiotics + BBR group and BBR alone group compared with that in the placebo and probiotics alone group (16). Berberine alters gut microbiota, microbiota-associated bile acid metabolism, and blood bile acid composition; it may exert hypoglycemic effects by inhibiting secondary bile acid production by *Ruminococcus bromii* (16).

The combination of BBR with other drugs is important in T2DM. The use of BBR (210 mg/kg), oryzanol (33.6 mg/kg), and vitamin B6 (7 mg/kg) for 4 weeks restores the relative abundance of Bacteroidaceae, Clostridiaceae in db/db mice. Bacteroidaceae and Clostridiaceae are considered to be bacteria that produce bile acid hydrolase, and the combination improves hyperglycemia. This effect may be connected to increased gut microbiota-mediated deoxycholic acid (DCA) production resulting in the upregulation of colonic TGR5 expression and glucagon-like peptide secretion, and improved glucose, lipid, and energy metabolism in db/db mice (17).

In addition, BBR (100 mg/kg, 150 mg/kg) and combined treatment with stachyose for 48 days can significantly improve glucose metabolism and reshape gut microbiota in Zucker diabetic fatty rats. Both BBR and BBR + stachyose have increased the abundance of *Akkermansiaceae, Erysipelotrich*aceae and Desulfovibrionaceae, and *decreased the abundance of Enterobacteriaceae*, *Christensenellaceae*, and *Bifidobacter*iaceae (18).

#### 3.1.2. Hyperlipidemia

Hyperlipidemia is a condition in which the level of fat in the blood [mainly total cholesterol (TC), triglycerides (TG) and low-density lipoprotein cholesterol (LDL-C)] is abnormally high (19). The prevalence of hyperlipidemia is rapidly increasing due to improvements in lifestyle and the popularity of high-calorie diets. It can suffer from an increased risk of causing various cardiovascular diseases (20). Various studies show that BBR has a good lipid-lowering activity; it significantly reduces TC, TG, and LDL-C concentrations and enhances serum high-density lipoprotein cholesterol (HDL-C)concentrations (1).

Berberine reduces blood lipids after 12 weeks of oral treatment (0.5 g, twice daily) in patients with hyperlipidemia. However, there were significant individual differences. BBR lowers cholesterol by regulating the gut microbiota. Baseline levels of *Alistipes* and *Blautia* accurately predict the anti-cholesterolemia effectiveness of BBR in subsequent treatment; the cholesterol-lowering effect of BBR is diminished in *Blautia*-deficient mice (21). Berberine also alters the intestinal microbial structure of rats on a high-fat diet., Species diversity and flora richness were markedly reduced after 4 months of intervention with BBR (150 mg/kg, orally). The abundance of *Christensenellaceae*, *Dehalobacteriaceae, Erysipelotrichaceae*, and *Peptococcaceae* (all Firmicutes) was significantly reduced (22). In addition, clinical studies have shown that nitroreductase (NR) from intestinal bacteria plays an important role in promoting intestinal absorption of BBR. Fecal NR activity is higher in patients with hyperlipidemia than in healthy individuals; blood BBR and fecal NR activity are positively correlated (23).

Berberine combined with probiotics improves postprandial hyperlipidemia in patients with T2DM. The effect of combined probiotic (with nine strains) and BBR treatment on postprandial lipids was assessed in 365 T2DM subjects (24). The combination of probiotics + BBR improves postprandial lipids (reduced TC, LDL-C, and multiple lipid metabolites) in patients compared with the treatment alone; this effect is associated with fecal *Bifidobacterium breve* enrichment; the presence of four fadD genes encoding long-chain acyl-CoA synthetase in *Bifidobacterium breve* strains. Berberine up-regulates fadD gene expression *in vitro* and further reduces the free fatty acid level in the culture medium. This may be the basis for probiotics + BBR to reduce the intestinal lipid uptake and blood cholesterol level of the host (24).

#### 3.1.3. Viscera injury related to glucose and lipid metabolism

The improvement of glycolipid metabolism also has a certain therapeutic effect on organ function impairment secondary to metabolic diseases. BBR can obviously promote the above metabolic processes, so it can ameliorate the organ function damage caused by metabolic related diseases to a certain extent. Ecological dysbiosis of gut microbiota is thought to underlie non-alcoholic steatohepatitis (NASH). The relative levels of *Bifidobacteria* and the proportion of *Bacteroidetes*: *Firmicutes* are restored in HFD-fed treated mice receiving BBR (200 mg/kg/d) by gavage for 8 weeks (25). Four weeks of intraperitoneal administration of BBR (150 mg/kg/d) alleviates HFD-induced hepatic steatosis and histopathological changes in the intestinal mucosa. The abundance of gut bacteria *Faecalibacterium prausnitzii* decreases and the abundance of *Bacteroides* increases (26). Also, BBR treatment of mice (100 mg/kg/d by gavage) for 4 weeks increases the relative abundance of *Clostridiale*s, *Lactobacillaceae*, and *Bacteroidales*, which mediates the activation of intestinal Farnesoid X receptor to alleviate NASH (27).

Berberine alters the gut microbiota environment in mice with alcoholic liver disease treated with BBR (10, 50, 100 mg/kg) by gavage, decreases the abundance of *Pseudoflavonifractor*, *Mucisirillum, Alistipes*, *Ruminiclostridium*, and *Lachnoclostridium* and increases the abundance of *Akkermansia muciniphila.* Among them, *Akkermansia muciniphila* is essential in maintenance of the gut barrier integrity and may induce the activation of specific cell subpopulations with immunosuppressive functions, thereby alleviating alcoholic liver injury (28).

Diabetes and hypercholesterolemia are high risk factors for atherosclerosis. Atherosclerosis (AS) is often the leading cause of cardiovascular disease due to the growth of connective tissue, deposition of intracellular and intracellular cholesterol, fatty acids, and calcium carbonate, accumulation of collagen and proteoglycan, hardening and thickening of artery walls, thinning of arteries, and loss of elasticity of the entire artery (29). In recent years, the incidence has been on the rise and is difficult to treat [(30, 31)]. BBR improves glycolipid metabolism through the regulation of gut microbiota, and at the same time improves the progression of atherosclerosis. Current studies have shown that gut microbiota is an influential factor in the development and deterioration of AS (32). BBR can change the intestinal microbial composition of mice *(Lachnospiraceae NK4A136 group, Bacteroidales S24-7 group) (unclassified)*, increased abundance of *Eubacterium*), and cutC/cntA gene abundance associated with trimethylamine (TMA) production; Under anaerobic conditions *in vitro*, BBR inhibits the formation of d9-TMA in a dose-dependent manner, and in mice, BBR can significantly reduce the elevated level of TMA-producing bacteria (33). Trimethylamine oxide (TMAO) is an independent risk factor and initiator of Atherosclerosis (34). Similarly, other studies show that BBR administration (50 mg/kg, twice weekly, by gavage) reduces the expression of TMAO and inflammatory cytokines with an increased abundance of Verrucomicrobia and decreased abundance of Firmicutes in BBR-treated mice (35). *Akkermansia* is an important bacterium in the Verrucomicrobia phylum; its abundance increases after 14 weeks of BBR (0.5 g/L) administration in the drinking water of ApoE^–/–^ mice. Meanwhile, BBR attenuates high-fat diet (HFD)-induced metabolic endotoxemia and reduces arterial and intestinal expression levels of inflammatory cytokines and chemokines. BBR attenuates metabolic endotoxemia caused by a high-fat diet (HFD) and reduces the expression of inflammatory and chemokines in the arteries and intestines, Anti-AS and metabolic management effects of BBR may be associated with increased abundance of *Akkermansia* (36). In conclusion, regulation of gut microbiota by BBR contributes to anti-AS, and BBR can be considered as one of the effective drugs for the treatment of AS.

### 3.2. Gastrointestinal disease

#### 3.2.1. Intestinal inflammatory disease

Inflammatory bowel disease (IBD) is a chronic inflammatory disease of the intestine of unknown origin, and its pathogenesis includes host genetics and immune response, gut microbiota, and environmental stimulation (37). Dysbiosis of gut microbiota is associated with IBD (38–40). Berberine (40 mg/kg for 7 days) alleviates dysbiosis in rats with dextran sodium sulfate (DSS)-induced colitis and significantly upregulates *Bacteroides* and *Akkermansia*; both animal and Caco-2 cell models show that BBR regulates gut microbiota by tryptophan metabolism and activation of the tryptophan receptor (AhR) pathway to improve the damaged intestinal barrier (41). Berberine may also prevent and treat ulcerative colitis by regulating intestinal microecology and protecting the intestinal mucosal barrier (UC) Berberine restores DSS-induced colonic inflammation by modulating intestinal microbes (42). It protects the colon by reconstructing the disrupted epithelial barrier, regulating the expression of immune factors, and enhancing the expression of the Wnt/β -catenin pathway. For DSS-induced colitis, the biological barrier was repaired after 7 days of treatment with BBR (40 mg/kg/d). Berberine increases the relative level of bacteria. Also, the expression of probiotic bacteria *Lactococcus* is upregulated compared to the model group, but the expression of conditionally pathogenic bacteria such asmouse intestinal *Bacteroides*, segmented filamentous bacteria, and *Enterobacteriaceae* decreases. Similar studies confirm that BBR increases the expression of lactic acid producing bacteria (*F. rodentium* and *Lactobacillus*) and carbohydrate hydrolyzing bacteria (*R. flavefaciens* and *B. pseudolongum*) and decreases the expression of conditionally pathogenic bacteria (*Mucispirillum*, *Oscillospira*, *B. uniformis*, and *Allobaculum)* to regulate the gut microbiota (43). In addition, BBR is significantly related to immune homeostasis in the gut. Berberine (100 mg/kg oral) regulates the differentiation of intestinal immune cells by affecting the growth of *Bacteroides fragilis* to relieve DSS induced colitis after 8 days of treatment (44).

#### 3.2.2. Irritable bowel syndrome

Irritable bowel syndrome (IBS) is a functional disorder of the intestine characterized by abdominal pain and abnormal bowel movements (45). IBS treatment focuses on a variety of causes, including changes to the gut microbiota, visceral hypersensitivity, intestinal permeability, and other factors that contribute to the disease’s pathophysiology (46). Patients with IBS have visceral hypersensitivity; this is thought to be related to the activity of spinal microglia. Berberine significantly alleviates chronic water avoidance stress-induced visceral hypersensitivity and reduces the activation of colonic mast cells and spinal microglia in rats (47). Berberine (200 mg/kg,14 days) does not directly inhibit LPS-induced microglia activation, but may inhibit it through the enrichment of SCFA-producing bacteria (*Anaerostipes*, *Eubacterium*, *Lachnoclostridium*, and *Eisenbergiella*).

Berberine has promising therapeutic effects on IBS in combination with other drugs. Berberine and baicalin (BA) form natural self-assemblies such as BA-BBR nanoparticles (BA-BBR NPs) and show synergistic effects on IBS-D. The 1:1 ratio of BA:BBR was mixed to form BBR-BA NPs and BA-BBR NPs (1.715 mg/d) followed by administration by gavage for 10 days. The relative abundance of Bacteroidia, Deferribacteres, Verrucomicrobia, Candidatus, Saccharibacteria, and Cyanobacteria was significantly remarkably higher in the IBS-D mouse model group than that in the normal group. However, BA-BBR NP treatment reduces the relative abundance of these phyla. BA-BBR NPs are most effective in treating visceral hypersensitivity and diarrhea in IBS-D model mice (48).

#### 3.2.3. Gastrointestinal tumor

Colorectal cancer (CRC) is one of the leading causes of cancer deaths worldwide (49). The ratio of intestinal flora plays a crucial role in the development of CRC (50). Berberine improves the tumor microenvironment by regulating the disturbed gut microbiota (51).

Oral administration of BBR (100 mg/kg for 10 weeks) to CRC mice significantly alters gut microbiota composition. Berberine inhibits pathogenic species such as *f_Erysipelotrichaceae* and *Alistipes* and increases the abundance of SCFA-producing bacteria including *Alloprevotella* and *Flavonifractor*. Also, metabolic data suggest that BBR can alter fecal metabolism by regulating the metabolism of sugars, amino acids and SCFA. These fecal metabolites are the product of the combined action of the host and the intestinal flora (52). The important role of SCFA bacteria was confirmed in another study. BBR significantly attenuates CRC progression and alters the gut microbiota structure in HFD-fed Apc ^min/+^ mice after 12 weeks of oral treatment with BBR (500 ppm). Berberine significantly inhibits the increase of Verrucomicrobia at the phylum level, inhibits *Akkermansia* at the genus level, and elevates the levels of SCFA-producing bacteria (*Lachnospiraceae*) (53). Recent studies show that BBR prevents p-azomethane (AOM)/DSS-induced CRC in mice by reducing inflammatory activation and improving intestinal flora dysbiosis. The release of inflammatory factors and cell proliferation markers is suppressed under BBR intervention (7.5 and 15 mg/kg), and key pathway proteins involved in the inflammatory process (p-STAT3 and p-JNK) and cell cycle regulatory molecules (β-catenin, c-Myc, and CylinD1) have lower expression levels, AOM/DSS stimulation results in a sharp decrease in abundance of beneficial bacteria, *Lactobacillus* and *Dubosiella*, and an increase in abundance of undesirable bacteria *Bacteroides*, *Escherichia, Shigella*, and *Akkermansia*. Meanwhile, the use of BBR restored the ratio of these bacteria to a relatively normal state (54). In conclusion, the anticancer effect of BBR is achieved through its regulation of the intestinal microbiota.

### 3.3. Liver disease

Liver disease is a life-threatening condition that includes liver fibrosis, cirrhosis, and drug-induced hepatotoxicity is one of the main reasons for mortality and morbidity all over the world. Hepatic fibrosis is a pathological marker and precursor of cirrhosis, and fibrosis occurs in relation to liver metabolism and gut microbiota homeostasis (55). It is suggested that gut microbiota can be an independent regulator of liver metabolism, affecting the fibrosis progression as well as the regression (56). Compared to normal mice, Germ-free mice show more severe signs of liver fibrosis (57). These studies suggest that dysbiosis of gut microbiota is the important driver of liver fibrosis. Berberine strengthens the endocrine capabilities of the gut microbiota, which further regulates the liver microenvironment and ameliorates fibrosis. The abundance of SCFA secreting bacteria increased due to berberine treatment (58). Short chain fatty acids are essentialin liver diseases. For example, butyrate alleviates inflammation and liver fibrosis by promoting anti-inflammatory cytokines including IL-4 and IL-10 and inhibits inflammatory genes such as TGF-β1 and IL-1α(59). In addition, BBR reduces hepatotoxicity caused by pathological or pharmacological interventions by improving the dysbiosis of gut microbiota (60).

Berberine has potential value in the treatment of liver diseases by reshaping the structure of the gut microbiota, especially by modulating the abundance of SCFA-producing bacteria (for example, *Clostridium* and *Bacillus*) and *Akkermansia muciniphila*.

### 3.4. Mental disorder

Berberine protects the *central* nervous system and has been shown to be effective in anti-depressant, anti-anxiety, and anti-inflammatory conditions. It reduces depressive and anxious behavior by suppressing neuroinflammation in mice under stress (61). The anxiety model of ovariectomized rats treated with BBR (100 mg/kg) for 4 weeks show significant improvement in anxious behavior and increased levels of the bacterial community metabolite equol (which has potential estrogen-like effects). These changes may be caused by an increase in beneficial bacteria such as *Bacteroides, Bifidobacterium*, *Lactobacillus*, and *Akkermansia* (62).

Berberine modulates gut microbiota and metabolic disturbances of patients with schizophrenia or bipolar disorder, as well as mild olanzapine-induced metabolic disturbances (63). For the patients with schizophrenia or bipolar disorder treated with olanzapine for at least 9 months, followed by 12 weeks of treatment with BBR (100–300 mg/tid), there is a remarkable decrease in the abundance of Firmicutes while a remarkable increase in the abundance of *Bacteroides*. Antipsychotic treatment can cause changes in the gut microbiota that induce chronic low-grade inflammation, suppress resting metabolic rates, and activate multiple signal transduction pathways, leading to metabolic dysfunction (64). Gut microbiota is promising for research of antipsychotic-induced metabolic disturbances, and BBR is a candidate for treatment.

### 3.5. Immune disease

Recent studies have shown that BBR has increasing importance in immune diseases. Graves’ disease is a multisystemic syndrome of autoimmune diseases (65). Berberine significantly upregulates the enterobactin synthesis and restores the thyroid function by increasing iron uptake. Methimazole alone did not affect the gut microbiota structure of patients alone, while combined treatment with BBR (0.3 g/three times a day) for 6 months significantly changes the flora structure of patients, increases the abundance of beneficial bacterium (such as *Lactococcus lactis*, and decreases the abundance of disease-causing bacteria (such as *Enterobacter hormaechei,Chryseobacterium indologenes*) (66). The gut microbiota of autoimmune uveitis mice is modified after 14 days of intragastric administration of BBR (100 mg/kg/d). Bacteria with immunoregulatory ability (such as *Lactobacillus*, *Akkermansia*, *Oscillibacter*, and *Ruminococcosaceae*) are enriched and play an important for immune homeostasis during autoimmune uveitis (67). Berberine (50 mg/kg,25 days) was used to treat acute graft-versus-host disease mice through gut microbiota remodeling (the abundance of Actinobacteria and Bacteroidetes and genus *Adlercreutzia*, *Lactobacillus*, *Dorea*, *Sutterella* and Plesiomonas were increased) and intestinal mucosal barrier protection, inhibition of TLR4 signaling pathway activation, and suppression of NLRP3 inflammatory vesicles and their cytokine release (68). In addition, another study demonstrates that BBR (200 mg/kg/d) inhibits CD8 + T_CM_ cells by reducing the abundance of *Bacillus cereus* to inhibit mouse islet allograft rejection (69). Berberine (200 mg/kg/d for 14 days) reduces collagen-induced arthritis (CIA) in rats by upregulating the relative abundance of intestinal SCFA-producing bacteria (*Blautia*, *Buttericicoccus*, and *Parabacteroides*) and significantly increases the content and proportion of butyric acid (70).

### 3.6. Other diseases

Oral BBR (100, 200 mg/kg) increases the amount of dopamine secretion in the brain to improve Parkinson’s disease (PD) symptoms by enhancing tyrosine hydroxylase activity in *Enterococcus* and promoting levodopa production in the intestine of a PD mouse model (71). Similar clinical findings show that oral administration of BBR (0.5 g, bid) for 8 weeks in 28 patients with hyperlipidemia increases the relative abundance of blood/fecal levodopa. Meanwhile, the relative abundance of *Enterococcus* increases by 11%, wherein *E. faecalis* and *E. faecium* are dominant. *Enterococcus* may synthesize dopa/dopamine in the gut, and BBR may promote dopa/dopamine levels *in vivo* through intestinal bacteria. On the other hand, berberine may have the same effect (71). Berberine treatment of osteoporosis in a ovariectomy-periodontitis rat model for 7 weeks (120 mg/kg) results in a significant increase in butyric acid-producing bacteria (*Blautia, norank_f_Bacteroidales_S24-7_group*, and *Roseburia*) compared to the control group, and the intestinal barrier integrity improves. Berberine treatment attenuates IL-17A-related immune responses in rats and reduces serum levels of pro-inflammatory factors; this suggests that BBR may treat periodontal bone loss caused by estrogen deficiency by regulating the gut microbiota (72).

## 4. Outlook

Gut microbiota can regulate the efficiency of BBR absorption and utilization *in vivo*; meanwhile, the structure and function of the gut microbiota will be changed due to the intervention of BBR (9). The effect of BBR on the gut microbiota varies depending on its dose (73). Good therapeutic effects on a variety of diseases can be achieved by the BBR-gut microbiota axis multi-target drug in future studies. However, BBR can cause therapeutic diarrhea, and the treatment-emergent mild diarrhea of BBR in normal rats is likely to be caused by ecological dysbiosis of gut microbiota (74). The pharmacological action and clinical research of BBR requires further investigation. Many preclinical experiments proved the role of BBR, and some clinical experiments also show good results. However, BBR has poor bioavailability. Therefore, attempts were made to use different dosage forms, drug delivery systems, and technologies such as microcapsules, nanoparticles, and other new drug carriers to improve its bioavailability and therapeutic effect. Future work should involve more clinical experiments to explore the mechanism of BBR-mediated gut microbiota regulating various diseases and accumulate more evidence to support its early intervention as a routine treatment.

## 5. Conclusion

This paper mainly describes the effects of different BBR doses on various diseases by regulating gut microbiota. The multi-pharmacological effect of BBR can be explained at least in part by its regulatory role in the gut microbiota. The evidences presented in this paper shows that the different roles of BBR in diseases are related to the diversity of gut microbiota. In diabetes, BBR plays a hypoglycemic role mainly through SCFA producing bacteria and bacteria related to bile acid metabolism. In patients with hyperlipidemia, BBR alters host lipid and cholesterol levels by regulating lipid synthesis related microbiota. In AS, BBR reduces the production of TMA, TMAO, inflammatory factors and chemokines by remodeling gut microbiota, thus playing an anti-AS role. In intestinal diseases, BBR mainly maintains the intestinal mucosal barrier, regulates immune homeostasis, reduces visceral hypersensitivity, and anti-inflammatory effects by altering lactic acid producing bacteria, carbohydrate producing bacteria, and SCFA producing bacteria. In the liver, BBR mainly alleviates liver damage by regulating SCFA producing bacteria and FXR related bacteria. In mental disorders, BBR plays an anti-inflammatory and anxiety relieving role by regulating microbiota related metabolites. In immune diseases, changes in the immune regulatory microbiota and SCFA producing bacteria in the body after BBR intervention play a role in regulating immunity and anti-inflammatory effects. But the effect of BBR on gut microbiota under pathological conditions seems to be quite different due to the large individual differences in the composition of gut microbiota. Moreover, there is a genetic gap between rodents and humans. Therefore, more advanced and large-scale clinical research is required in order to investigate the impact of BBR on the regulation of the gut microbiota under pathological conditions.

## Author contributions

DX and RL conceived the study and took responsibility for the integrity of the study. FY, RG, and XL joined in study design, data analysis, data interpretation, manuscript preparation, and revised the manuscript. All authors contributed to the intellectual content of the manuscript and approved the final version submitted for publication.
